# La cardiothyréose au centre hospitalier universitaire de Bobo-Dioulasso, Burkina Faso

**Published:** 2012-03-06

**Authors:** Aimé Arsène Yaméogo, Nobila Valentin Yaméogo, Yves Daniel Compaoré, Tinoago Laurent Ouédraogo, Patrice Zabsonré

**Affiliations:** 1 Service de Cardiologie, Centre Hospitalier Universitaire Souro Sanou, Bobo-Dioulasso, Burkina Faso; 2 Institut Supérieur des Sciences de la Santé/Université Polytechnique de Bobo-Dioulasso, Burkina Faso; 3 Service de cardiologie, Centre Hospitalier Universitaire Yalgado Ouédraogo, Ouagadougou, Burkina Faso; 4 Unite de Formation et de Recherche-Science De la Santé (UFR-SDS), Université de Ouagadougou, Burkina Faso

**Keywords:** Hyperthyroïdie, Cardiothyréose, Burkina-Faso

## Abstract

**Introduction:**

La cardiothyréose est une affection fréquente et grave à l’ouest du Burkina Faso. Notre objectif était d’étudier les caractéristiques épidémiologiques, cliniques, thérapeutiques et évolutives des cardiothyréoses au centre hospitalier universitaire de Bobo-Dioulasso.

**Méthodes:**

Etude prospective de 12 mois pourtant sur des cas de cardiothyréose colligés dans les services de cardiologie et de médecine interne.

**Résultats:**

Quatorze (14) cas de cardiothyréose ont été colligés soit 33,3% des patients hospitalisés pour hyperthyroïdies. L’âge moyen des patients était de 53,57 ans ± 9,97. Les femmes au foyer (71,40%) aux conditions socio-économiques défavorables étaient les plus touchées. Parmi nos cas 21,42% des patients avaient déjà un antécédent d’hyperthyroïdie et l’HTA était le facteur de risque cardiovasculaire majeur (64,28%). Tous les patients présentaient une insuffisance cardiaque associée à un trouble du rythme (57,14%), essentiellement à type de fibrillation auriculaire (42,9%), une insuffisance coronarienne (7,14%) et un trouble de la conduction (7,14%). Le goitre multi-nodulaire a été l’entité étiopathogénique la plus fréquente (57,10%). Les antithyroïdiens de synthèse, les mesures hygiéno-diététiques et un traitement spécifique de l’insuffisance cardiaque ont été constamment utilisés pendant une durée d’hospitalisation moyenne de 23,57 jours ± 7,54. Si l’évolution immédiate peut être satisfaisante avec une euthyroïdie à 28,5% à moyen terme, les ruptures thérapeutiques peuvent être mortelles (un patient soit 7,14%) chez des patients généralement âgés majoritairement de sexe féminin avec un niveau socio-économique bas.

**Conclusion:**

Le traitement de la cardiothyréose est efficace d’où l’intérêt d’une politique sanitaire pour une prise en charge adéquate.

## Introduction

Le Burkina-Faso est l’une des régions les plus affectées par le goitre endémique au monde [1]. En dépit de la prévention accrue de l’endémie goitreuse, on assiste à l’émergence de cas d’hyperthyroïdie. Si ceux-ci sont relégués au second plan des priorités sanitaires à cause des pathologies infectieuses, en cardiologie, elles constituent toujours une préoccupation majeure du fait de ses complications cardiaques. En effet, le diagnostic de cardiothyréose repose sur l’association d’une thyrotoxicose à une atteinte cardiaque à type d’insuffisance cardiaque, d’insuffisance coronarienne, de troubles du rythme ou de la conduction [2]. Les travaux sur la cardiothyréose sont peu fréquents en Afrique et pour la plupart rétrospectifs. Aussi cette étude prospective avait pour but d’évaluer la prévalence et les difficultés de prise en charge de cette affection particulièrement dans la partie ouest du Burkina-Faso.

## Méthodes

Nous avons mené une étude transversale sur une année. Il s’agissait d’étudier du 1^er^ Janvier au 31 Décembre 2008, les aspects épidémiologiques, cliniques, thérapeutiques et évolutifs des cardiothyréoses au centre hospitalier universitaire de Bobo-Dioulasso.

Ont été inclus dans cette étude, tous les patients des deux sexes âgés de plus de 16 ans reçus en consultation externe ou hospitalisés et traités pour cardiothyréose dans le service de cardiologie et de médecine interne du centre hospitalier universitaire Souro Sanou de Bobo-Dioulasso. Le diagnostic de cardiothyréose a été retenu devant des signes cliniques d’hyperthyroïdie, confirmés à la biologie par une élévation de T4 totale ou T4 et ou de T3 totale ou T3 libre avec effondrement de TSH, associés au moins à l’une des atteintes cardiaques suivantes : insuffisance cardiaque, insuffisance coronarienne, troubles du rythme ou de la conduction.

Ont été exclus, les suspicions cliniques d’hyperthyroïdie sans confirmation biologique et les cas d’hyperthyroïdie sans signe d’atteinte cardiaque.

La collecte des données a été faite sur une fiche de recueil comportant les données cliniques (interrogatoire, signes physiques, et les aspects évolutifs) et para cliniques. Les définitions opérationnelles étaient: L’HTA pour une pression artérielle = 140/90mmHg selon l’OMS de 1999; L’obésité par un index de masse corporelle (IMC = P/T^2^)>30; La cardiomégalie radiographique (ICT>0,50) et l’hypertrophie ventriculaire gauche électrocardiographique pour un indice de SOKOLOW >35mm; L’hyperthyroïdie a été retenue pour les taux: de T3 libre>6ng/L (= 9pmol/L) et/ou T3 totale>2,8nmol/L; Soit de T4 libre>19,5ng/L (=25pmol/L) et/ou T4 totale>168nmol/L; Associés à TSHUS<0,25mUI/L ou non ultrasensible<0,4mUI/L.

Au plan évolutif : une consultation de suivi a été réalisée à 1mois, 4mois et un an après la mise du patient sous antithyroïdiens de synthèse. La carbimazole (Néomercazole^®^) a été la molécule utilisée. Les critères appréciés ont été : Au plan clinique : la régression des signes de thyrotoxicose, la prise de poids, l’amélioration et la stabilisation de l’état hémodynamique, la survenue d’une récidive de décompensation cardiaque ou de décès; Au plan biologique, la normalisation du taux des hormones thyroïdiennes avec : TSHUS entre 0,25-6mUI/L et non ultrasensible entre 0,4-9mUI/L; T4 Libre entre 7,5-19,5ng/L (9,5-25pmol/L) et T4 total entre 51-168nmol/L; T3 libre entre 2-6ng/L (3-9pmol/L) et T3 totale entre 1,5-2,8nmol/L. Les données ont été saisies et analysées à l’aide du logiciel Epi info version 3.5.1.

## Résultats

Du 1^er^ Janvier au 31 Décembre 2008, 2323 patients ont été vus dans le service de cardiologie (1786 consultants et 537 hospitalisés) et 4617 en Médecine interne (2597 consultants et 2020 hospitalisés). Quarante-deux (42) patients ont présentés une hyperthyroïdie biologiquement confirmée. Des signes de complication cardiaques ont été retrouvés chez 14 d’entre eux, soit 33,3% des hyperthyroïdies.

### Caractéristiques des patients

L’âge des patients variait entre 39 et 71 ans avec un âge moyen de 53,57 ans ± 9,97. 42,9% des patients avaient entre 49 et 59 ans. Une prédominance féminine a été notée : 13 femmes (92,9%) contre un seul Homme soit un sex-ratio de 1/13. Il s’agissait surtout de femmes au foyer (10 cas soit 71,40%, dont 5 veuves). Une seule patiente était fonctionnaire toujours en activité. La plupart des patients provenait du milieu urbain de Bobo-Dioulasso (11cas soit 78,6%). Les 3 autres patients (21,42%) provenaient des provinces rurales avoisinantes.

Les facteurs de risque cardiovasculaires retrouvés étaient : une HTA retrouvée chez 8 patients soit 57,14% des cas; une surcharge pondérale chez 2 patientes (14,28%) et une obésité de type androïde chez une patiente (7,14%). Trois patientes étaient déjà porteuses d’une hyperthyroïdie.

### Les types d’atteintes cardiaques

**Au plan clinique:** Le délai moyen de dépistage des nouveaux cas de cardiothyréose chez nos patients était de 30,45 mois±28,01 mois. Les circonstances de découverte étaient représentées par : la dyspnée 12 cas (85,71%), les œdèmes des membres inférieurs 10 cas (71,42%), l’amaigrissement, les palpitations respectivement chacune 5 cas (35,71%) et les précordialgies 4 cas (28,57%). La répartition des patients selon le type d’atteinte cardiaque est présentée sur le Tableau 1. Une insuffisance cardiaque globale a été retrouvée chez l’ensemble des patients, associée ou non à une insuffisance coronarienne secondaire, à un trouble du rythme ou de la conduction à type de bloc de branche droit complet. Il s’agissait dans les sept cas d’une arythmie complète par fibrillation auriculaire. L’insuffisance cardiaque était classée stade II (trois cas soit 21,42%), stade III (neuf cas soit 64,28%) et stade IV (deux cas soit 15,38%). L’examen physique des patients a retrouvé : une HTA associée dans 64,28% (9 cas), un signe de Harzer dans 64,3% (9 cas), une insuffisance mitrale fonctionnelle 57,1 (8 cas), une insuffisance tricuspidienne fonctionnelle dans 28,6% (4 cas), un galop gauche dans 35,7% (5 cas) et un galop droit dans un cas (7,1%).

**Tableau 1:** Répartition des cas de cardiothyréose selon l’atteinte cardiaque

Types d’atteintes cardiaques	Effectifs	Pourcentages
Insuffisance cardiaque globale	14	100
Insuffisance coronarienne	1	7,14
Trouble du rythme	8	57,14
Tachycardie Sinusale	6	42,9
Extrasystole ventriculaire isolée	1	7,14
Trouble de la conduction	1	7,14

**Au plan para clinique:** L’analyse de l’ECG des patients a révélé l’existence d’une hypertrophie ventriculaire gauche chez 12 patients soit 85,7% des cas et des troubles du rythme surtout, avec respectivement la fibrillation auriculaire et la tachycardie sinusale respectivement 50% et 42,9%. L’échographie thyroïdienne réalisée chez l’ensemble des patients retrouve un goitre multi nodulaire chez huit patients (57,1%), un aspect compatible avec une maladie de Basedow dans cinq cas (35,7%) et un infiltrat tissulaire d’allure maligne chez une patiente (7,1%). Une élévation des taux de thyroxine qu’elle soit libre ou totale a été régulièrement associée à une thyrotoxicose tandis que la tri-iodo-thyronine (T3 libre ou Totale) était élevée dans 71,42% des cas. Le Tableau 2 montre la répartition des patients selon les résultats des dosages des hormones thyroïdiennes.

**Tableau 2:** Répartition des patients selon l’hormonologie thyroïdienne

Hormones thyroïdiennes Normes	Valeurs moyennes	Valeurs extrêmes	Effectif (>Norme)	Effectif (<Norme)	Effectif Total
TSH: 0,25-6mUI/L	0,012	0,00-0,05	---	14	14
T4 libre: 7,5-19,5 ng/L	62,78	50,98-82,62	6	---	6
T3 libre: 2-6 ng/L	10,02	1,52-30,50	2	1	5
T4 totale: 51-168 nmol/L	249,22	188,51-320	8	---	8
T3 totale: 1,5-2,8 nmol/L	5,31	2,99-7,74	8	---	8

**La prise en charge des patients:** Des mesures hygiéno-diététiques ont été nécessaires chez l’ensemble des patients de même que la prise d’un antithyroïdien de synthèse. La molécule utilisée était la carbimazole. Les autres médicaments utilisés sont présentés sur le Tableau 3.

**Tableau 3:** Les moyens thérapeutiques misent en œuvre

Moyens thérapeutiques	Effectifs	Pourcentages
Mesures hygiéno-diététiques	14	100
Antithyroïdien de synthèse (Carbimazole)	14	100
Bêtabloquants (Propranolol ou Aténolol)	10	71,4
Vasodilatateurs (Dérivés nitrés et ou IEC)	13	92,9
Diurétiques	13	92,3
Digitaliques	7	50
Antiagrégants plaquettaires	12	85,7
Anxiolytiques	3	21,4

### Les aspects évolutifs

**Figure 1:**
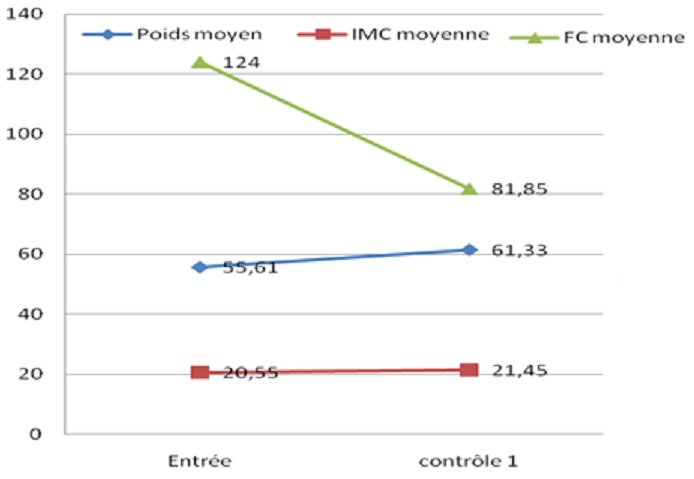
Evolution des constantes après un mois de traitement

Une hospitalisation initiale a été nécessaire chez sept patients soit 50% des cas. La durée moyenne de l’hospitalisation était de 23 jours ± 7,5 (extrêmes 14 à 37 jours). L’évolution a été favorable pour l’ensemble des patients hospitalisés avec la régression totale des signes d’insuffisance cardiaque et une stabilisation de l’état hémodynamique avec une régularisation du rythme cardiaque. A un mois, l’évolution clinique était favorable chez tous les patients. La stabilité de l’état hémodynamique et de la fréquence cardiaque était associée à un gain pondéral et à une régression de la thyrotoxicose (Figure 1). L’évolution ultérieure à quatre mois a été marquée par quatre ruptures thérapeutiques (28,57%) en rapport avec la faiblesse de revenus de nos patients. Ces ruptures ont occasionnée une reprise évolutive de deux cas de thyrotoxicose ayant nécessité une hospitalisation d’une patiente de 41 ans et de 71 ans. Elle sera fatale chez cette dernière soit un taux de létalité de 7,14%. Au bout d’un an, seul quatre patients présentaient une euthyroïdie, soit 28,57% de notre effectif.

## Discusiion

### Aspects épidémiologiques

La cardiothyréose est une affection fréquente au Burkina Faso et particulièrement dans la région Ouest. En effet 33,3% des hyperthyroïdies se sont compliquées d’une cardiothyréose au centre hospitalier universitaire Souro Sanou de Bobo-Dioulasso. Drabo [1] avait déjà noté en 1996, une prévalence de 22,2% à Ouagadougou. Cette prévalence importante est supérieure à celle de la littérature sous régionale qui est de 15% à Conakry [3], 14% à Abidjan [4], 10,52% au Sénégal [5]. Elle reste en concordance avec les données de Heulin [6], qui estimait que la cardiothyréose complique 10 à 60% des hyperthyroïdies. Valcke [2] en France trouvait 20% et Ben Mrad, 16% à Sfax en Tunisie [7]. Si la cardiothyréose est une affection ubiquitaire, l’absence de dosage hormonal du fait d’une insuffisance de ressources financières chez de nombreux patients suspects d’hyperthyroïdie est un facteur limitant le diagnostic. Du reste, les caractéristiques sociodémographiques montrent que les errances diagnostiques de la cardiothyréose sont source d’un diagnostic tardif confirmant l’âge des patients comme premier facteur prédisposant dans notre série et dans la plupart des études [7–10]. La prédominance féminine est classique [9,11,12] et même parfois exclusive comme dans l’étude de Lokrou [4]. Elle pose le problème de l’accessibilité financière aux soins. En effet 71,40% des patients de notre série étaient des femmes au foyer, issues de milieux socioéconomiques défavorisés et dépendantes donc directement des revenus de leurs époux. Les patients provenaient à 78,6% d’un milieu urbain mettant ainsi en exergue les difficultés de diagnostic dans les zones rurales mais aussi l’inaccessibilité financière et géographique aux centres de références. En effet seuls trois patients provenaient des provinces avoisinantes (Léraba, Ioba et Banwa); ceci n’est qu’une partie visible de l’iceberg.

### Aspects Diagnostiques

**Les antécédents et les motifs de consultation:** L’hypertension artérielle a représenté, dans notre série, l’affection cardiovasculaire sous-jacente la plus fréquente (57,10%). Elle s’est révélée d’ailleurs plus fréquente que dans les séries de Nkoua [13] au Congo (30%) et de Diallo [14] (20%). Dans les séries de Drabo [8] et de Niakara [10], dans la région du centre du Burkina, l’HTA était associée à la cardiothyréose respectivement dans 31,1% et 25%. Cette disparité pourrait s’expliquer par la diversité des modes de recrutement, la plupart de ces études étant rétrospectives. Cependant il semble important de noter que la prévalence de l’HTA est en constante progression, ce qui rend compte de sa forte proportion dans notre série, plus récente. Seules deux valvulopathies ont été observées. Wade [5] à Dakar avait fait le même constat. Il s’agissait d’un cas de rétrécissement aortique et d’un cas de prolapsus de la grande valve mitrale. Le prolapsus de la grande valve mitrale paraît classique dans la cardiothyréose. Diallo [14] au Mali décrivait deux cas de prolapsus de la valve mitrale contre un cas chez Wade [5]. Un antécédent d’hyperthyroïdie a été retrouvé chez 3 de nos patients, soit 21,42%, taux proche de celui rapporté au Mali (26%) [14].

### Le tableau clinique

Le polymorphisme clinique de la cardiothyréose [3,9,14], mais aussi le coût élevé des dosages des hormones thyroïdiennes sont une importante source d’erreurs diagnostiques, bien que la plupart des cas soient diagnostiqués à un stade caricatural (92.9% des patients présentaient une thyrotoxicose typique faite de palpitations, de précordialgies, d’amaigrissement et de thermophobie). En effet, les délais de dépistage sont très longs, 30,45 mois de délai moyen de dépistage dans notre série, 34 mois selon Ben Mrad [7] à Sfax et 20,14 mois à Abidjan selon Lokrou [4].

**Les types étio-pathogéniques de la thyrotoxicose:** Le goitre multi-hétéro-nodulaire a été l’entité étio-pathogénique la plus retrouvée dans notre série, suivie de la maladie de Basedow et un cas de suspicion de cancer thyroïdien (7,10%). Cette distribution diffère de celle habituellement retrouvée dans la littérature où la prédominance de la maladie de Basedow est classique. En fait, une grande hétérogénéité caractérise les séries. Dans les régions du centre du Burkina Faso, Drabo [8] avait observé 51,4% de maladie de Basedow, 28,6% de goitre multi-hétéro-nodulaire et 17,1% de nodule toxique. Niakara [10] et Diallo [14] avait trouvé la même tendance. La proportion de goitre multi-nodulaire et de Maladie de Basedow ne représentait que 40,0% dans la série de Nkoua [13] à Brazzaville. A Abidjan, Renambot [12] notait une proportion équivalente entre Maladie de Basedow et adénome toxique (40%) tandis qu’une prédominance du goitre multi nodulaire à l’origine d’une cardiothyréose est rapportée par d’autres auteurs Africains [15,16] et Français [17].

**Les manifestations cardiaques:** L’insuffisance cardiaque est fréquente dans les cardiothyréoses. Valke [2] dans sa revue générale l’estimait entre 4 et 50% avec une moyenne de 10%. Des prévalences plus importantes sont rapportées en Afrique [5,8,10]. D’emblée globale, elle a été retrouvée chez l’ensemble de nos patients comme dans la plus part des séries [5,13].

Les troubles du rythme: une fibrillation auriculaire est largement décrite au cours des cardiothyréoses. Elle est en effet présente chez 2 malades sur cinq selon la littérature sous régionale. [9,10,14]. Elle est très fréquemment associée à une insuffisance cardiaque, cependant cette double association est très variable dans la littérature, 75% dans la série de Wade [5] contre seulement 14,30% chez Drabo [8] et 42,85% dans notre série.

L’insuffisance coronarienne: d’énormes disparités caractérisent la prévalence de l’insuffisance coronarienne dans la littérature. En effet si Valke [2] dans une revue générale sur la cardiothyréose situe la proportion attendue d’insuffisance coronarienne entre 0,5% et 20%, les séries de Niakara [10] et Drabo [8] trouvaient respectivement à Ouagadougou 25% et 18,03% largement en deçà des données de Lokrou [4] à Abidjan (42,8%). Notre série n’a retrouvé que 7,14% et Renambot [12] à Abidjan et Nkoua [13] à Brazzaville n’avaient d’ailleurs pas retrouvé de cas. Dans tous les cas il semble qu’une coronarographie soit plus que nécessaire pour confirmer les diagnostics au regard de la sensibilité et de la spécificité de l’électrocardiogramme.

Les troubles de la conduction sont très peu retrouvés dans les publications africaines [5,8,14]. Drabo [1,8] n’a noté que 8,2% sur 61 cas colligés en 9 ans et 10% sur 10 cas en 4 ans. Notre travail, avec un cas de bloc de branche droit incomplet (7,1%), retrouve les mêmes tendances. Aucun cas de BAV n’a été noté dans notre série.

### Aspects thérapeutiques

Le traitement a été essentiellement médical dans notre série. Il a fait appel à une hospitalisation initiale dans la moitié des cas, avec un séjour hospitalier moyen de 27,57 ± 7,54 jours. Cette durée moyenne d’hospitalisation est supérieure aux 14,1 jours de Diallo [14] au Mali et 18,3 jours retrouvés à Ouagadougou par Niakara [10].

Ce séjour prolongé en milieu hospitalier s’expliquerait d’une part, par le retard à l’initiation du traitement du fait de la nécessité d’un diagnostic de certitude par les dosages hormonaux avant la mise en route d’un traitement, et d’autre part, par la sévérité du tableau clinique présenté par les patients. La carbimazole du fait de son efficacité déjà prouvée [8,10] a été l’antithyroïdien de synthèse de choix. Sa tolérance a été bonne dans l’ensemble avec un seul cas de leucopénie modérée et transitoire n’ayant pas nécessité l’arrêt du traitement. La chirurgie n’a pas été effective dans notre étude, à cause du délai de suivi relativement court de 12 mois. La prise en charge de l’insuffisance cardiaque dans la cardiothyréose a été classique. Elle a fait appel aux mesures hygiéno-diététiques et à un traitement digitalo-diurétique associé aux vasodilatateurs et aux bêtabloqueurs. Il s’agit d’un schéma thérapeutique utilisé par la plupart des auteurs. L’HTA a été traitée par des inhibiteurs de l’enzyme de conversion ou des inhibiteurs calciques en fonction de l’état hémodynamique de patients. Dans notre étude, le traitement de la fibrillation auriculaire a reposé sur les bêtabloqueurs seuls ou associés à des digitaliques. Il a fait appel de façon systématique aux antiagrégants plaquettaires pour prévenir les complications thromboemboliques assez fréquentes dans la cardiothyréose [9,10]. Les antiagrégants plaquettaires ont été préférés du fait de leur coût plus abordable, contrairement aux anticoagulants de manipulation plus délicate pour des malades illettrés avec des contraintes financières pour les dosages de TP-INR de contrôle des antivitamines K (AVK). Aucun cas de thrombose n’a été constaté. La fibrillation auriculaire a été réduite dans les 6 cas (100%). Ce taux est supérieur aux 53% rapporté par Niakara [10] à Ouagadougou.

### Aspects évolutifs

L’évolution de la cardiothyréose dans notre série, diffère peu des résultats décrits dans la littérature. L’évolution immédiate a été favorable avec une stabilisation de l’état hémodynamique constatée chez tous les malades revus (100%). Ces données concordent avec celles de Diallo [14] au Mali et de Niakara [10] au Burkina, qui avaient obtenu respectivement 94% et 97%. Cependant comme toute pathologie chronique, de nombreuses ruptures thérapeutiques ont jalonnés l’évolution à court et moyen termes. En effet, 85,71% des patients avaient présenté au moins une rupture d’approvisionnement un mois après le début du traitement. Ce taux est passé à 44,44% au quatrième mois de suivi et était nul au dernier contrôle entre le huitième et le douzième mois.

L’insuffisance d’observance du traitement est imputable, dans 88,8% des cas, à l’accessibilité financière aux médicaments notamment à la carbimazole. Cependant, ce taux se réduit considérablement lorsque les malades ont véritablement pris conscience de la chronicité de leur maladie. Les deux cas de reprise évolutive du syndrome de thyrotoxicose, de même que la persistance d’une thyrotoxicose biologique 4 à 6 mois après l’initiation du traitement témoignent de la nécessité d’une bonne observance. La reprise évolutive de la thyrotoxicose surtout quand elle survient chez un patient d’âge avancé a un mauvais pronostic. Dans notre série elle a été fatale chez une patiente de 71 ans soit une létalité de 7,14%. Diallo [14] au Mali rapportait 6% durant les 3 premiers mois de suivi et Drabo [8] 5% avec un recul de 22 mois.

Les complications notamment thromboemboliques décrites dans la littérature n’ont pas été mises en évidence dans notre série.

Au plan biologique, le monitorage hormonal indispensable au suivi des patients constitue une difficulté majeure dans la prise en charge des patients dans notre contexte de travail du fait des coûts qui sont très onéreux et du nombre de contrôles à réaliser. 28,57% de nos patients ont présenté une euthyroïdie biologique après un an, alors que Drabo [8] en 2003 rapportait 14,75%. Il semble donc que l’usage exclusif de la carbimazole assure un taux élevé de succès thérapeutique. En effet, au regard de la persistance de signes de thyrotoxicose sous benzylthiouracil, après 3 mois de traitement, une substitution par la carbimazole avait été nécessaire dans les séries de Niakara [10] et Drabo [8].

## Conclusion

La cardiothyréose de par sa morbidité et sa létalité, constitue un problème de santé publique dans la région sud-ouest du Burkina Faso. Elle touche essentiellement une tranche fragile de la population constituée de femmes âgées, inactive et sans revenue pour leur prise en charge. Si le diagnostic est facilement évoqué à la clinique, le coût élevé des dosages hormonaux et du traitement est un handicap majeur de la prise en charge. Le traitement antithyroïdien associé au traitement spécifique de l’atteinte cardiaque a suffisamment prouvé son efficacité. Une bonne politique sanitaire axée sur les personnes âgées visant à faciliter le diagnostic précoce et à amoindrir les coûts de prise en charge serait donc très bénéfique.
